# mRNA Lipid Nanoparticles for Cell Engineering in Vivo and in Vitro: Current Applications and Future Directions

**DOI:** 10.1002/mco2.70700

**Published:** 2026-05-10

**Authors:** Lina Li, Menglan Wang, Xiuhan Ye, Zhiyan Liu, Yijing Duan, Xiaoming Chen, Yue Ouyang, Qibiao Wu, Mengjuan Sun, Tian Xie

**Affiliations:** ^1^ Faculty of Chinese Medicine and State Key Laboratory of Mechanism and Quality of Chinese Medicine Macau University of Science and Technology Macao China; ^2^ School of Pharmacy Hangzhou Normal University Hangzhou Zhejiang China; ^3^ College of Medicine Jinhua University of Vocational Technology Jinhua Zhejiang China; ^4^ Liangzhu Laboratory Zhejiang University Zhejiang Hangzhou China; ^5^ Zhuhai M.U.S.T. Science and Technology Research Institute Guangdong‐Macao Ln‐Depth Cooperation Zone in Hengqin Zhuhai Guangdong China; ^6^ Center For Clinical Pharmacy, Cancer Center, Department of Pharmacy Zhejiang Provincial People's Hospital (Affiliated People's Hospital), Hangzhou Medical College Hangzhou China

**Keywords:** cell engineering, lipid nanoparticles, mRNA technology, targeted delivery

## Abstract

Messenger RNA‐lipid nanoparticles (mRNA‐LNPs) serve as a revolutionary platform, enabling precise and transient protein expression for both in vivo and in vitro cell engineering without genomic integration. Recent breakthroughs in mRNA design and LNP formulation have expanded their applications across immunotherapy, regenerative medicine, and genome editing. However, challenges such as off‐target delivery, immunogenicity, and inadequate organ‐specific targeting limit their broader therapeutic utility. This review systematically elaborates the design principles of mRNA‐LNPs, including mRNA structural elements and functional lipid components that facilitate endosomal escape. It summarizes recent advances in their applications for cell engineering, both ex vivo and in vivo. Key challenges related to delivery precision and immunogenicity are thoroughly analyzed, alongside strategies to improve targeting through administration routes, surface modifications, and endogenous targeting mechanisms. The article also outlines main directions for developing next‐generation mRNA‐LNPs. Overall, this review will support further research on mRNA‐LNPs and promote their clinical translation in the field of cell engineering.

## Graphical abstract

Schematic diagram of mRNA‐lipid nanoparticles (mRNA‐LNP) and its functional mechanisms, applications, and challenges in cell engineering. This figure details the structural composition of mRNA‐LNPs and the delivery strategy, highlighting three core challenges. It further visualizes the key steps in the delivery pathway, from cellular uptake through endosomal escape to the subsequent translation of the mRNA into interest protein within the cytoplasm.

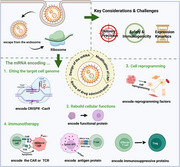

## 1 Introduction

**FIGURE 1:**
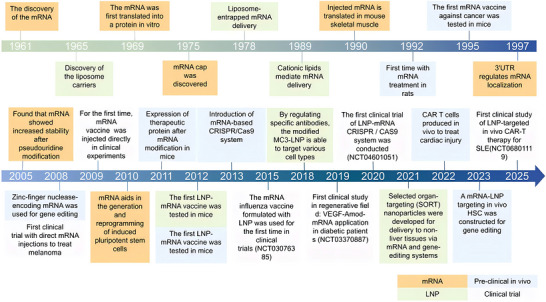
Event schedule for the mRNA‐LNP. This timeline covers the technological breakthroughs and key experimental milestones of mRNA‐LNP technology from its discovery to clinical application since 1961. HSC represents hematopoietic stem cell. Car‐T represents chimeric antigen receptor T.

Cell engineering has become a cornerstone of modern therapeutics, enabling disease treatment through precise regulation of cellular functions. The emergence of messenger RNA‐lipid nanoparticle (mRNA‐LNP) technology represents a paradigm shift in the field, allowing transient and programmable protein expression without genetic modification [1, 2]. Over the past decade, rapid advancements in this technology have reshaped treatment strategies for cancer, genetic disorders, and regenerative medicine. The development process of mRNA technology primarily encompasses four key aspects: (1) the discovery and modification of mRNA; (2) ongoing advancements in delivery vehicles that enable mRNA to effectively navigate physiological barriers and target specific cells; (3) preclinical research of various mRNA therapeutic approaches; and (4) the successful outcomes of mRNA technology in clinical trials, which have significantly heightened public interest and accelerated its popularization and commercialization (Figure 1).

Despite the potential of mRNA technology, its practical application within the body encounters several challenges, including inherent immunogenicity, expression stability, and precise cellular targeting [3]. Achieving effective delivery of mRNA to target cells requires overcoming a series of physiological barriers, wherein delivery carriers are crucial for ensuring efficient mRNA expression. Recent research has focused on developing innovative materials and novel formulation strategies to address this issue. Among these, lipids have emerged as one of the most successful materials for promoting mRNA delivery due to their inherent biocompatibility [4]. Fortunately, numerous studies have shown that through high‐throughput screening and rational design, LNPs exhibit significant potential in enhancing delivery efficiency and targeting accuracy [5, 6]. LNPs have already been successfully applied in clinical practice, particularly in the development of mRNA‐based COVID‐19 vaccines [7]. Although LNPs have been approved for the treatment of human diseases, challenges remain regarding their transient expression kinetics, immune activation, and scalable manufacturing. Therefore, there is an urgent need for a systematic review that integrates recent advances in mRNA‐LNP design, mechanisms of action, and clinical applications to address the pressing demand for a comprehensive analysis in this rapidly evolving field.

This review begins by introducing the core components of mRNA‐LNPs, including optimized mRNA structures and functional lipids, and explains their key roles in maintaining stability, enabling endosomal escape, and achieving targeted delivery. It then explores the mechanisms of cellular uptake and intracellular trafficking. Furthermore, the article systematically surveys recent advances in both in vitro applications, such as CAR‐T cell engineering, dendritic cell (DC) vaccine preparation, and stem cell manipulation, and in vivo applications, including protein replacement therapy, genome editing, and tissue reprogramming. Special emphasis is placed on innovative strategies to enhance delivery precision, such as administration routes, surface functionalization, and logic‐controlled nanoparticles (NPs). Finally, the review provides an in‐depth analysis of persistent challenges in safety, immunogenicity, and expression control, while outlining future directions involving artificial intelligence (AI)‐assisted LNP design, scalable production, and room‐temperature stabilization. These advances pave the way for developing safe, effective, and accessible cell engineering therapies based on mRNA‐LNP technology.

## 2 mRNA‐LNP Design and Mechanisms

As an emerging method for delivering biological macromolecules, mRNA has demonstrated its significance in the vaccine sector [8]. Although RNA is more prone to degradation and less stable compared with DNA, it offers the advantage of reduced off‐target effects due to the absence of permanent insertion. Recently, mRNA‐based technology has increasingly become the focal point of drug development.

### 2.1 Core Components and Optimization

The mRNA‐LNP system adopts a “core–shell” architecture. Its components are designed to protect mRNA integrity, enable targeted delivery, and trigger intracellular release, working synergistically to ensure delivery efficiency and biosafety. The specific constituents and optimization strategies are outlined below.

#### 2.1.1 mRNA Molecular Design

The key components of mRNA include the 5′ cap, untranslated regions (UTRs), coding sequence (CDS), and the 3′ poly(A) tail. The 5′ end incorporates an m^7^GpppG Cap‐1 structure to facilitate ribosome recognition. Short UTRs derived from α‐globin or β‐globin can be selected to enhance translation initiation efficiency and mRNA stability—for instance, the α‐globin 5′ UTR increases the translation efficiency of luciferase mRNA by 2.5‐fold [9]. Within the CDS, codon optimization (e.g., replacing rare codons) and reduction of uridine content help minimize Toll‐like receptor (TLR)3/7‐mediated innate immune activation [10]. As an example, mRNA containing N1‐methylpseudouridine (m1Ψ) reduces IFN‐α secretion in DCs by 80% compared with unmodified mRNA [11]. Furthermore, the introduction of a 120–150 nt poly(A) tail at the 3′ end extends the intracellular half‐life of the mRNA [12].

#### 2.1.2 LNP Components and Their Functions

LNPs primarily consist of an ionizable cationic lipid (CIL), a helper phospholipid, cholesterol, and a PEGylated lipid (Table 1). The functions of each component are as follows:

**TABLE 1:** Anti‐CD19 responses of mRNA‐based CAR‐cells.

Year	Chimeric antigen receptor type	Target	Delivery	References
2020	CAR‐T	CD19	C14‐4; LNPs in vitro	[13]
2022	CAR‐T	CD19	LNP in vitro	[14]
2022	CAR‐T	CD19	76‐O17Se; LNPs in vitro	[15]
2022	CAR‐M	CD19	9322‐016; LNPs in vitro	[15]

The CILs serve as the core functional component for mRNA compaction and endosomal escape. Its molecular structure features a protonable amine headgroup and hydrophobic alkyl tail(s). At neutral physiological pH (7.4), the CIL remains neutral, reducing nonspecific binding with plasma proteins. Upon entering acidic endosomes (pH 5.0–6.0), the amine headgroup becomes protonated, acquiring a positive charge. This enables, on one hand, electrostatic compaction of the mRNA to maintain complex stability, and on the other hand, interaction with anionic phospholipids in the endosomal membrane, disrupting membrane integrity and facilitating escape [16, 17]. For instance, the clinically used DLin‐MC3‐DMA (approved for COVID‐19 mRNA vaccines) can transition from a disordered inverse micellar phase (LII) to an inverse hexagonal phase (HII) at pH 5.5, directly disrupting the endosomal membrane via this structural change [16].

The helper phospholipid, typically distearoylphosphatidylcholine (DSPC), forms the main structural framework of the LNP shell. Its saturated fatty acyl chains contribute to a rigid membrane structure, enhancing stability and reducing mRNA leakage during circulation [18]. DSPC also works synergistically with the CIL to modulate membrane curvature, promoting membrane fusion or rupture in the acidic endosomal environment. For example, at a 1:4 molar ratio of DSPC to CIL, the endosomal escape efficiency of LNPs can reach 35%, significantly higher than the 12% observed without DSPC [19].

Cholesterol is embedded within the lipid gaps of the LNP shell. It modulates the membrane fluidity imparted by CILs, preventing LNP aggregation during circulation. Cholesterol also helps reduce the cytotoxicity associated with CILs; for instance, LNPs containing cholesterol showed a 40% decrease in liver injury markers (ALT/AST) in mice compared with cholesterol‐free formulations [20].

The PEGylated lipid, often DSPE‐PEG2000, is anchored on the LNP surface. The PEG chains form a hydrated layer that sterically hinders phagocytosis by the reticuloendothelial system, prolonging the circulation half‐life to over 6 h (compared with only 1.5 h for non‐PEGylated formulations) [21, 22]. Furthermore, PEGylation helps control particle size and dispersity, inhibits lipid aggregation, and confines the LNP size typically within the 50–100 nm range [23].

### 2.2 Cellular Uptake Mechanisms

**FIGURE 2:**
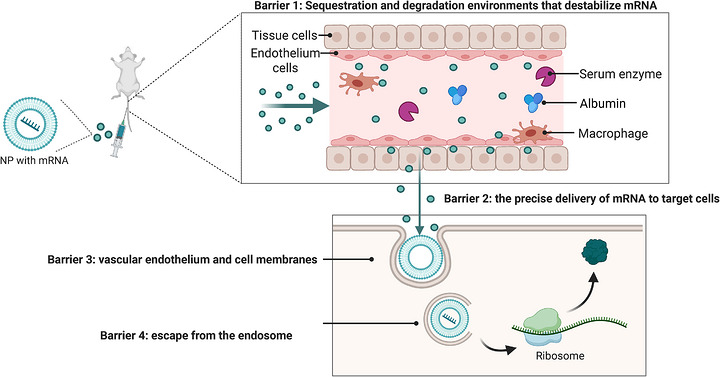
The main barriers for mRNA‐based delivery. The four main barriers mRNA faces during in vivo delivery: sequestration and degradation in the biological environment, precise targeting, penetration of the endothelium and cell membranes, and effective release.

Overcoming various physiological obstacles, enhancing the safety and efficiency of mRNA delivery, and achieving in vivo engineering of target cells are among the key challenges in this field. These physiological barriers include both extracellular and intracellular challenges, primarily including (i) sequestration and degradation environments that destabilize mRNA, (ii) difficulties in the precise delivery of mRNA to target cells, (iii) barriers of vascular endothelium and cell membranes, and (iv) obstacles associated with intracellular endosomes (Figure 2). The specifics are outlined as follows.

### 2.3 Sequestration and Degradation Environments That Destabilize mRNA

Blood and extracellular fluid contain various enzymes and immune‐related molecules, including serum ribonucleases that hydrolyze RNA, as well as organ‐specific resident macrophages that sequester mRNA from the bloodstream. Furthermore, cells deploy multiple mechanisms to combat foreign nucleic acid molecules [24]. Naked RNA is unstable and requires chemical modifications and effective delivery strategies for in vivo mRNA delivery. These modifications safeguard RNA from degradation by extracellular nucleases and prevent sequestration by recognition receptors. Examples include substitutions like 5‐methylpyrimidine, N1‐methyladenosine, and so on, on the ribose, alterations to internucleotide bonds, 3′‐end capping, and the use of locked nucleic acids with a methylene bridge [25, 26]. Due to the inherent instability of mRNA, the use of mRNA conjugated to an appropriate carrier that can protect it is preferred over the administering naked mRNA. Despite being shielded from harsh chemical environments by protein or lipid carriers, these vectors still face encounter challenges from the immune system. The complement system can target some vectors, while antibody recognition can result in macrophage clearance, reducing effectiveness [27]. Thus, it is essential to develop advanced delivery systems that can endure harsh conditions and exhibit minimal immunogenicity.

### 2.4 Barriers of Vascular Endothelium and Cell Membranes

In vivo delivery of mRNA‐carrier is challenging due to their size (>5 nm) preventing endothelial barrier penetration and their negative charge repelling cell membranes, making it hard to diffuse into target tissue and cross cell membranes, unlike small molecules (≤100 Da). Although viral vectors have inherent mechanisms to surmount cellular barriers, they are often linked to significant immune responses and serious side effects [28, 29, 30, 31]. Consequently, well‐designed and manufactured nonviral vectors are increasingly favored. Nonviral vectors primarily facilitate transmembrane transport through endocytosis [32]. Optimizing this endocytosis process can involve various strategies, such as employing binding proteins that correspond to membrane receptors or modifying the composition, size, shape, and charge of the carrier. Notably, the modification of the targeting antibody and its binding to the receptor may influence internalization [33]. The internalization rate seems to be positively correlated with payload release [34, 35]. However, predicting these rates in vivo remains challenging, prompting efforts to develop in vitro systems that replicate the process effectively [36, 37].

### 2.5 Obstacles Associated With Intracellular Endosomes

After cellular uptake, an ideal delivery vehicle should effectively escape from endosomes and release its cargo into the cytosol for further intracellular processing [38, 39]. Studies indicate that insufficient endosomal unpacking speed of multimeric complexes compromises transfection efficiency [40]. Currently, four primary mechanisms of endosomal escape are widely discussed: (i) *membrane fusion mechanism*: ensures fusion within endosomes to avoid nontargeted fusion [41]. (ii) *Osmotic rupture mechanism*: as the endosome acidifies, polymers with buffering capacity can prevent a further decline in pH, prompting cells to continue pumping protons into the endosome. This process triggers chloride ions influx, resulting in an increase in internal pressure and ultimately leading to lysis [42]. It may trigger various unintended side effects, primarily focusing on cellular stress [43], immune activation, and organelle damage [44]. (iii) *NP swelling mechanism*: the phenomenon of NP swelling has been observed in various delivery processes; however, its specific impact remains a subject of debate [28, 45, 46]. (iv) *Membrane destabilization mechanism*: used in polymer‐based NPs to destabilize endosomes membrane for escape. The acidic environment of the endosome can be leveraged to induce structural changes in the carrier, thereby facilitating endosomal escape and the release of cargo [45, 47].

## 3 Current Applications: In Vitro Cell Engineering

In vitro cell engineering involves the qualitative modification of the genetic and physiological functions of ex vivo cells, enabling precise regulation of their proliferation, differentiation, metabolism, and other activities to obtain desired engineered cells for therapeutic, research, or production purposes. Currently, significant progress has been made in areas such as immunotherapy, regenerative medicine, and disease modeling. However, challenges remain, including operational complexity and imprecise functional outcomes in target cells. Conventional viral vectors pose risks of genomic insertion, while methods like electroporation and nano‐perforation cause substantial cell damage. Additionally, the CRISPR–Cas9 system faces issues such as off‐target effects and delivery challenges, creating a pressing need for new technologies. Recently, mRNA‐LNP technology has gained attention due to its nonintegrating nature, which avoids genomic risks, its flexibility for multiplexed gene editing via combined mRNA delivery, and its simple procedures coupled with rapid, highly efficient expression. Current trends indicate that this technology is progressively replacing traditional methods. In the following sections, we will discuss in detail the application of mRNA‐LNP technology in cell engineering and immune cell programming in vitro.

### 3.1 Cell Engineering of CAR Therapy

CAR‐T and CAR‐M cells enhance their ability to recognize and eliminate specific tumor antigens by expressing chimeric antigen receptors (CARs). The success of CAR‐T therapy has inspired the development of CAR applications in other immune cell types. The conventional production of CAR‐T cells involves multiple steps, including T cell isolation, activation, genetic engineering, expansion, and reinfusion [48]. In current clinical studies, electroporation remains the standard method for delivering CAR‐encoding mRNA into T cells. This technique uses electrical pulses to temporarily disrupt the cell membrane, allowing mRNA entry. However, irreversible membrane damage and loss of intracellular components can reduce cell viability and alter gene and protein expression [49], which may explain the suboptimal performance of mRNA‐based CAR‐T cells in clinical trials. To overcome the limitations of electroporation, LNPs offer a promising alternative for mRNA delivery in CAR‐T cell engineering. As fully synthetic carriers, LNPs exhibit lower cytotoxicity and may enable more efficient production of mRNA‐based CAR‐T cells. Comparative studies have shown that LNP‐derived CAR‐T cells outperform those generated by electroporation in terms of CAR mRNA delivery efficiency, CAR expression persistence, and functional durability in vitro. Moreover, LNP‐engineered CAR‐T cells achieved comparable in vitro function to CAR‐T cells produced via lentiviral transduction, supporting LNPs as a competitive nonviral platform for cell engineering [50]. Billingsley et al. from the University of Pennsylvania have validated LNPs as an effective mRNA delivery platform for T cell engineering [14]. The team developed a synthetic mRNA delivery vector named C14‐4 LNPs specifically for CAR‐T cell modification [13]. In a separate study, chemically modified CAR mRNA was successfully delivered to primary macrophages and CD8^+^ T lymphocytes using two distinct LNP formulations: 9322‐O16B and 76‐O17Se [15]. Most of these investigations represent preclinical studies, with short‐term anti‐CD19 responses of mRNA‐based CAR‐cells summarized in Table 1. Collectively, these findings establish LNPs as a next‐generation mRNA delivery platform for in vitro CAR‐T cell engineering, providing a solid foundation for preclinical evaluation in mouse models and future clinical translation.

### 3.2 Tumor Vaccine and DC Activation

DC therapy operates on the principle of loading patient‐specific tumor antigens into DCs, which are then reinfused into the body. These antigen‐presenting DCs activate T cells by presenting tumor antigens, thereby stimulating an immune response that leads to cancer cell apoptosis. In a study by Zhang et al., total tumor‐derived RNA was encapsulated in LNPs to target DCs, demonstrating that LNP‐packaged tumor RNA can function as an effective antigen‐specific vaccine [51]. Another study proposed a combined strategy termed CATCH, which integrates mRNA‐LNP formulations with DC therapy. This approach significantly enhances the reprogramming of the tumor microenvironment (TME) and amplifies T cell activation. The study included a comparative analysis between adoptively transferred CD40‐BMDCs and the activation of endogenous DCs [52], offering a novel perspective for improving experimental reliability.

### 3.3 Regulation of Immune Suppressive Cells

With the growing maturity of autoimmune cell therapies, treatment strategies centered on Foxp3^+^ regulatory T cells (Tregs) have garnered significant attention. This specialized T cell subset, known for its immunomodulatory properties, offers a promising therapeutic pathway for reestablishing immune balance through its unique suppressive functions. In one study, researchers developed an LNP‐based platform to deliver a variant of Foxp3 mRNA to CD4^+^ T cells in vitro, generating immunosuppressive Foxp3‐T (iTreg) cells for the treatment of autoimmune diseases [53].

### 3.4 Stem Cell and Progenitor Cell Manipulation

Stem cells possess considerable potential for ex vivo therapy due to their capabilities of self‐renewal and differentiation, which contribute to tissue homeostasis and regeneration [54]. Recent breakthroughs in regenerative medicine and disease research have involved directing the differentiation of stem cells into specific functional cell types using LNP technology. One study reported the directed differentiation of patient‐derived induced pluripotent stem cells into dopaminergic neurons [55], resulting in functional dopamine (DA) neurons for Parkinson's disease (PD) cell therapy. Kim et al. established an mRNA‐based gene delivery method for the generation of safe DA neurons in vitro [56]. Successful application of LNP technology in this mRNA delivery approach could offer new possibilities for producing healthy and safe therapeutic cells. Additionally, another study demonstrated that LNPs carrying siRNA can induce the osteogenic differentiation of mesenchymal stem cells (MSCs) in vitro [57], confirming the utility of LNPs for genetic modification in bone marrow‐derived MSCs. The transport and delivery capabilities of LNPs may also extend to other bone marrow‐resident cells, opening new avenues for in vitro stem cell differentiation, disease modeling, drug screening, and potential cell transplantation therapies with improved efficiency and convenience.

### 3.5 Organoid or Tissue Engineering

Several studies have incorporated small molecule conjugates into LNP formulations to enhance targeting specificity and cellular uptake efficiency. For instance, the small molecule ligand streptavidin has been applied in vitro to improve transfection across different cellular compartments. Moreover, mRNA‐LNP technology has been successfully utilized in diverse areas such as gene editing, cell reprogramming, tissue regeneration, and dynamic regulation. In disease modeling via gene editing, LNPs delivering CRISPR–Cas9 mRNA can precisely introduce disease‐associated mutations into organoids, enabling the construction of human pathological models in vitro. For example, Wei et al. demonstrated the potential of LNP‐mediated delivery of CRISPR–Cas9 mRNA [58]. Although organoids were not explicitly mentioned in that study, the application of CRISPR–Cas9 gene editing in organoids is well established [59, 60], providing technical support for subsequent organoid engineering. In recent years, integration of LNP and mRNA technologies has also led to advances in vascularization therapies within tissue engineering. For instance, LNP‐delivered VEGF mRNA has been shown to promote the formation of functional lymphatic networks after myocardial infarction [61]. In vitro, similar strategies could be applied to 3D‐bioprinted skin tissues to facilitate vascular network formation and improve graft survival, offering new solutions for ischemic tissue repair and skin transplantation.

## 4 Current Applications: In Vivo Cell Engineering

Based on the aforementioned targeting strategy, the delivery efficiency of mRNA drug to target cells can be significantly enhanced. This improvement is essential for achieving treatments that necessitate high levels of protein expression, such as protein replacement therapies and in situ programming of CAR therapy. In the following sections, we will discuss the advancements in mRNA drug‐targeted therapies within these domains.

### 4.1 Targeted Reprogramming in Disease Models

Building on the advances in mRNA delivery systems described in previous sections, targeted reprogramming represents a transformative approach for in vivo cell engineering. Unlike conventional cell therapies that require ex vivo manipulation, this strategy aims to directly modify the identity and function of endogenous cells within their native microenvironment. The core objective is to convert existing cell types into desired functional states, thereby replacing lost or dysfunctional cells without the need for cell transplantation. Two primary mechanistic approaches have emerged in this field: protein replacement therapy for restoring specific molecular functions, and genome editing for permanent correction of genetic defects. Both strategies leverage the tissue‐specific targeting capabilities of advanced LNP systems to achieve localized therapeutic effects while minimizing systemic exposure.

#### 4.1.1 Protein Replacement

Protein replacement therapy involves substituting or supplementing specific defective proteins with functional proteins that are either absent or mutated in affected patients, thereby restoring their functionality. This therapeutic approach encompasses methods such as recombinant protein production and gene therapy. While the direct delivery of recombinant proteins can circumvent the cellular translation process, it presents challenges including the protein's short half‐life, instability, and immunogenicity, rendering it unsuitable for replacing intracellular proteins. In comparison with protein or DNA therapy, mRNA therapy offers significant advantages. It can circumvent processes such as nuclear insertion and transcription, is considered safer, fails to incorporate into the host genome, leading to temporary effects and consequently minimizing the potential for mutagenesis. Consequently, mRNA‐based protein replacement therapy emerges as a promising treatment modality with extensive applications across various diseases, including lung diseases [62], cardiovascular conditions [63], hematological disorders [64], cancers [65], metabolic disorders [66], neurogenic disorders [67], orthopedic diseases [68], and muscle atrophy. Currently, numerous clinical trials are researching the use of mRNA techniques for protein replacement in specific diseases (Table 2).

**TABLE 2:** The clinical application of protein replacement and cell reprograming in vivo.

Drug name	Condition	mRNA	Delivery system	Administration	Results	Status	NCT number
AZD8601	Heart failure	VEGF‐A	Naked mRNA	Epicardial injection	Seven individuals who had coronary artery bypass graft surgery experienced a safe and well‐tolerated recovery, with no adverse events or detrimental effects noted throughout the 6‐month follow‐up duration [69].	Phase II; completion	NCT03370887
	Male subjects with Type II diabetes			Intradermal	Basal cutaneous blood flow was well tolerated, and mild injection site reactions was the only treatment‐related adverse event [70].	Phase I; completion	NCT02935712
MRT5005	Cystic fibrosis	Human cystic fibrosis transmembrane regulator protein (CFTR)	LNP	Inhalation	Pulmonary function remained stable after the treatment, but no benefit was observed. Treatment was generally safe and well tolerated, with fever and hypersensitivity resolving rapidly in some subjects [71].	Phase I/II; recruiting	NCT03375047
mRNA‐3927	Propionic acidemia	Alpha and beta subunits of propionyl‐CoA	LNP	Intravenous infusion	The mid‐term results are encouraging and are well tolerated by the patients [72].	Phase I/II; recruiting	NCTO4159103
ARCT‐810	Ornithine transcarbamylase deficiency	Ornithine transcarbamylase	LNP	Intravenous infusion	Not yet	Phase Ia/Ib/II; completed, terminated	NCT04416126/NCT04442347/NCT05526066
mRNA‐3704	Methylmalonic acidemia	Methylmalonyl‐coenzyme A mutase (MUT)	LNP	Intravenous infusion	Unknown	Phase I/II; withdraw	NCT03810690

*Data source*: https://clinicaltrials.gov/.

Cystic fibrosis, a genetic disorder resulting from mutations in the CFTR gene, can have its function significantly restored through the transfection of CFTR mRNA. In animal studies, intranasal injection of LNPs‐CFTR mRNA has been shown to restore up to 55% of the net chloride ion efflux characteristics [73]. Additionally, the mRNA‐based CFTR protein MRT5005 has progressed to the clinical research stage [74].

In the context of hematological diseases, mRNA therapy demonstrates considerable potential. Hemophilia, a bleeding disorder caused by a deficiency of coagulation factors, can benefit from protein replacement therapy achieved by delivering the appropriate coagulation factors via mRNA. In a hemophilia mouse model, FVIII and FIX mRNA encapsulated in LNPs can rapidly induce and sustain the expression of these crucial coagulation factors, significantly enhancing the coagulation function of the mice [75, 76].

In the field of metabolic diseases, mRNA therapy has garnered significant attention. Currently, there are no effective treatments for rare genetic metabolic disorders such as liver and kidney tyrosinemia and acute intermittent porphyria. However, delivering the relevant enzyme genes via mRNA can restore enzyme activity and thereby improve the condition. For instance, in mice with liver and kidney tyrosinemia, fumarylacetoacetate hydrolase mRNA delivered by LNPs can significantly enhance liver function [77]. In mice with acute intermittent porphyria, LNP‐encapsulated mRNA can induce the expression of human porphyria, with cholinogen deaminase restoring normal urinary porphyrin precursor excretion [78]. Furthermore, metabolic diseases such as methylmalonic acidemia and Fabry disease have also demonstrated the effectiveness of mRNA therapy [79, 80, 81].

mRNA therapy also plays an active role in tumor treatment. The PTEN gene, recognized as an effective tumor suppressor, is often deleted or mutated in various cancers. The delivery of the PTEN gene via mRNA can significantly inhibit the prostate cancer [82]. Similarly, p53 mRNA has been shown to induce apoptosis and inhibit the liver cancer and non‐small cell lung cancer [83]. Furthermore, mRNA encoding antiangiogenic proteins can effectively suppress the growth of pancreatic tumors [84].

#### 4.1.2 Genome Editing Therapy

mRNA is widely utilized for delivering programmable nucleases [85]. These nucleases have demonstrated efficient transfection and manipulation of insertion/deletion mutations in the form of mRNA. Gene‐edited expression of mRNA poses no risk of mutation, making it an attractive method for gene‐editing therapy. Most current clinical trials of gene editing in vitro necessitate that the target cells, extracted from the organism, undergo gene editing in a controlled laboratory environment prior to being reinjected into the organism [86]. This approach allows for precise control over the editing process and may be particularly advantageous for diseases that involve specific cell types [87]. However, this method is intricate and requires considerable time, and ensuring that the edited cells will operate properly after reintroduction poses significant challenges. In contrast, in vivo delivery offers simplicity, as gene modification occurs directly within the organism, potentially enhancing treatment efficacy for a variety of diseases, particularly systemic diseases [88]. The rapid development of LNP has facilitated their widespread use for in vivo delivery of various gene editing agents. Currently, several clinical trials involving in vivo mRNA gene editing are underway (Table 3). However, most of these trials target liver delivery with liver tropism for its LNP as mentioned above. Therefore, more advanced strategies are needed to achieve efficient and safe nonliver delivery. Dahlman et al. [88] successfully achieved organ‐targeted delivery of the mRNA‐based CRISPR/Cas9 system by screening hundreds of LNPs. Additionally, the anti‐CD117 antibody‐conjugated LNPs developed by Laura Breda [89] can specifically target various mRNA‐encoded gene editing agents to hematopoietic stem cells (HSCs). It is anticipated that with the advent of new materials and innovative targeting methods, personalized, cell‐specific gene editing can be realized through mRNA‐based in vivo delivery.

**TABLE 3:** The clinical application of gene editing with mRNA in vivo.

Drug name	Condition	Target gene	Delivery system	Administration	Results	Status	NCT number
NTLA‐2001	Transthyretin amyloidosis	Transthyretin (TTR)	LNP; CRISPR/Cas9	Intravenous infusion	Use of NTLA‐2001 caused only minor adverse events and resulted in decreased serum TTR protein concentration by targeted knockdown of TTR [90].	Phase III; recruiting	NCT06128629; NCT04601051
NTLA‐2002	Hereditary angioedema	KLKB1	LNP; CRISPR/Cas9	Intravenous infusion	A single dose of NTLA‐2002 produced a significant, dose‐dependent, and durable reduction in total plasma kinin release levels, and no serious adverse events were observed [91].	Phase I/II; active, not recruiting	NCT05120830
YOLT‐201	Transthyretin amyloidosis	transthyretin (TTR)	LNP; CRISPR/Cas9	Intravenous infusion	Not yet	Phase I; recruiting	NCT06082050
VERVE‐101	Familial hypercholesterolemia and cardiovascular disease	PCSK9	LNP; CRISPR/Cas9	Intravenous infusion	Not yet	Phase Ib; completed	NCT05398029
VERVE‐102	Familial hypercholesterolemia and cardiovascular disease	PCSK9	GalNAc‐LNP; CRISPR/Cas9	Intravenous infusion	Not yet	Phase Ib; recruiting	NCT06164730

*Data source*: https://clinicaltrials.gov/.

### 4.2 Regenerative Approaches

The goal of this strategy is to stimulate or supplement existing cells, promoting their proliferation and differentiation to repair damaged tissues. It primarily targets tissue‐resident progenitor cells, stem cells, or functional somatic cells with inherent proliferative capacity. By delivering mRNA encoding growth factors, cytokines, or transcription factors, endogenous repair pathways are activated, which enhances cell proliferation and guides differentiation toward desired cell types. Applications include cardiac repair after myocardial infarction (by stimulating cardiomyocyte proliferation), skin wound healing, bone regeneration, and angiogenesis. This approach closely mimics natural physiological repair processes, offering potentially higher safety and relying on relatively mature technological strategies. However, it requires precise control over the extent of proliferation and differentiation to avoid excessive tissue growth, such as fibrosis or tumor formation. Furthermore, the efficiency and duration of mRNA delivery must be carefully matched to the kinetics of the tissue repair process.

Direct reprogramming, also known as transdifferentiation, refers to the alteration of cell fate. The introduction of cell reprogramming factors can facilitate the transformation of cells into the desired phenotype, thereby aiding in the repair of damaged tissue [92]. For instance, the delivery of four transcription factors—TBX5, MEF2C, HAND2, and GATA4 —into proliferating noncardiomyocytes in mice effectively transforms these cells into functional myocytes resembling cardiac cells [93]. However, the use of retroviral vectors or DNA can result in the integration of the delivered genes into the genome, often leading to oncogenic transformation, which poses challenges for subsequent clinical applications. In contrast, mRNA‐LNP technology effectively addresses this issue. Numerous studies have highlighted the potential of mRNA to reprogram cells and promote tissue regeneration [94] (Table 4).

**TABLE 4:** The cell reprograming via mRNA in vivo.

Reprograming factors	mRNA	Function	References
Transcription factors	PUMA	Promote hematopoietic stem cells differentiation	[95]
	RUNX1	Repair of the degenerative neurons	[96]
	HNF4A	Reversal of liver fibrosis and cirrhosis	[97]
Growth factors	VEGF‐A	Promote the conversion of bile duct epithelial cells into hepatocytes	[98]
	HGF, EGF	Reverse liver injury	[99]
	VEGF‐A	Stimulate the regeneration of ischemic cardiomyocytes; and enhance vascular growth	[100, 101, 102]
	TGF‐β	Promote lung endothelial cell regeneration	[103]
Others	BDNF	Protection of the ischemic brain neurons	[104]
	BMP‐2	Stimulate bone regeneration	[105, 106]

Currently, a significant number of transcription factors delivered via mRNA‐LNP have successfully achieved in vitro cell reprogramming and show promise for application in cell therapy [107]. Although in vivo evidence remains limited, some studies suggest the potential for reprogramming cells in vivo through the delivery of transcription factor mRNA. Recent reports indicate that mRNA encoding the p53 upregulated modulator of apoptosis (PUMA), when encapsulated in LNPs, can facilitate HSCs differentiation in vivo [95]. Lin et al. targeted the treatment of degenerative disc disease by directly injecting modified mRNA encoding the transcription factor RUNX1 into the intervertebral disc [96]. Additionally, the injection of HNF4A mRNA‐LNP in mouse models has been shown to alleviate liver fibrosis and cirrhosis [97]. However, it is important to note that differences may exist in the transcriptional circuits governing reprogramming between humans and mice, which could limit the applicability of these findings in human subjects.

Applying growth factor‐encoding mRNA to damaged cells represents an effective strategy for restoring cell phenotype and function. Given the liver tropism of LNP, this approach holds significant promise in the treatment of liver diseases. Specifically, mRNA‐LNPs encoding VEGF‐A has been shown to enhance liver function in vivo by facilitating the reprogramming of bile duct epithelial cells into hepatocytes [98]. In a mouse model of chronic liver injury, the injection of hepatocyte growth factor and epidermal growth factor mRNA‐LNPs rapidly reversed steatosis and promoted liver regeneration following acute liver injury, ultimately leading to the recovery of liver function [99]. Furthermore, there are also many studies in the cardiovascular field. mRNA therapy encoding VEGF‐A has been shown to stimulate postischemic myocardial regeneration, enhance blood vessel growth [100], and increase blood vessel density in both the skin and heart of animal models. Additionally, this approach has been found to improve cardiac function in pigs with experimental myocardial infarction [101, 102]. Presently, this therapy is in clinical development and is being tested in the AstraZeneca‐sponsored EPICCURE Phase 2a clinical trial for patients with moderately impaired left ventricular function undergoing surgical revascularization (NCT03370887). Furthermore, it has been reported that mRNA‐LNPs encoding TGF‐β can regenerate the lung endothelial phenotype, thereby facilitating the repair of lung damage following viral infection [103].

Additionally, other various reprogramming factors have also been applied in vivo. For instance, Fukushima et al. sought to protect brain tissue from neuronal death induced by ischemic attacks through the intracerebroventricular injection of BDNF mRNA [104]. Furthermore, BMP‐2 mRNA‐LNPs have been shown to promote bone regeneration in mouse models of femoral [105] and calvarial [106] defects.

These studies illustrate the considerable potential of mRNA technology in the realm of cell reprogramming. However, the duration and targeting of mRNA drugs require further optimization to maximize reprogramming efficacy while minimizing side effects. Cells from different‐aged donors vary in reprogramming efficiency, leading to genomic instability in pluripotent stem cells [108, 109]. Thus, it's crucial to study patient adaptability to in vivo reprogramming and set criteria for efficacy and safety.

### 4.3 Immune System Redirecting

#### 4.3.1 In Situ Programing of CAR Therapies in Vivo

In situ CAR cell therapy represents an innovative approach to directly edit a patient's immune cells within their body. This therapy focuses on the systemic delivery of CAR‐expressing mRNA or engineered viral vectors encoding CAR to immune cells in situ, enabling these cells to express specific surface proteins that target and attack disease cells. This method eliminates the need for the complex and time‐consuming ex vivo processing traditionally associated with cell therapies. Currently, both LNPs and polymer–NPs can effectively deliver CAR‐encoding nucleic acids into T lymphocyte [110, 111, 112, 113, 114, 115] and macrophages [116, 117]. CAR constructs with shorter half‐lives, such as mRNA‐based CAR T cells, offer a means to achieve transient and controlled CAR expression, presenting a potential avenue for more manageable treatments. Parayath et al. successfully developed an anti‐CD8 modification‐polymer‐based carrier‐loaded CAR mRNA complex that generated CD19‐specific CAR/TCR‐T cells in vivo, demonstrating an average sustained receptor expression for 7 days [111]. In comparison with virus‐induced T cells in vitro, engineered CAR/TCR‐T cells exhibited similar antitumor efficacy against hepatocellular carcinoma, prostate cancer and lymphoma [111]. Rurik et al. created CD5‐targeted fibroblast activation protein CAR mRNA‐LNPs to generate CAR‐T cells in vivo for the treatment of heart fibrosis [112]. Similarly, Epstein et al. [118]. conjugated anti‐CD5 antibodies to the LNPs, enabling the specificity delivery of CAR‐encoding mRNA to T lymphocyte, which resulted in the repair of heart damage in mice. Notably, the interaction between various RNA components, such as the codelivery of PD‐1 siRNA and CD19 CAR mRNA, enhances the action effect to T cells compared with the use of RNA alone [119]. DESCARTES‐08, a novel therapy developed by Cartesian, represents the first new approach for CAR‐T cells (rCAR‐T) utilizing mRNA‐LNPs to treat autoimmune diseases, and has now progressed into clinical trials for the first time. The results from the Phase 1b/2a clinical study indicate that DESCARTES‐08 has the potential to reduce the symptoms of myasthenia gravis in a more durable manner and is well tolerated, with no significant adverse reactions reported in patients [120].

With the successful application of in vivo CAR‐T therapy, researchers are now exploring a broader spectrum of cell types. Monocytes and macrophages have emerged as candidates for CAR modification due to their robust tissue infiltration and immune regulatory functions. In vivo CAR‐M therapy, which involves CAR‐modified monocytes or macrophages, has demonstrated significant therapeutic potential. The intraperitoneal administration of CAR‐M resulted in a significant enhancement of the adaptive immune response in two mouse models with syngeneic solid tumors, and it significantly enhanced the effectiveness of PD‐1/PD‐L1 immune checkpoint blockade in models resistant to treatment [121]. Mukalel et al. designed oxidized mRNA‐LNPs with a natural tropism for monocytes to engineer CAR‐monocytes in vivo, demonstrating enhanced antitumor effects in mouse experiments [122]. Recently, LNPs were utilized to generate CAR‐Macrophages in vivo for the treatment of cardiac fibrosis, further expanding the therapeutic potential of this platform [123]. The field of in vivo induced CAR‐T/M cell therapy remains in its infancy, facing challenges in clinical application, such as off‐target CAR delivery and systemic infusion of NPs [47]. Following systemic infusion, off‐target CAR delivery can induce unintended immune responses due to complement activation by the protein corona, anti‐PEG antibody reactions, or recognition by PRRs [124, 125]. The heterogeneity of the tumor EPR effect and remodeling of the protein corona can lead to nonspecific accumulation of CAR‐LNPs in organs such as the liver and spleen [126]. This accumulation poses a risk of damaging hepatocytes and macrophages, while the endosomal escape mechanism may further disrupt the structural integrity of these off‐target cells [43]. Overall, these risks must be comprehensively managed by combining LNP characteristics with in vivo behavior analysis to enhance the safety of CAR delivery. However, compared with traditional in vitro engineering, the utilization of mRNA technology for the instantaneous production of CAR‐immune cells in vivo is cost effective, time efficient, and promising.

#### 4.3.2 Vaccine

In recent years, mRNA vaccines are recognized as promising therapies due to their inherent advantages, and they are considered as safe and effective strategies for the diseases prevention and treatment [127]. Mechanistically, mRNA vaccines deliver a synthetic template of the antigenic protein into target cells via in vivo delivery. Subsequently, these antigenic proteins are degraded and presented to MHC Class I or MHC Class II complexes, which are then recognized by T cells or B cells, thereby triggering cell‐mediated adaptive immune responses or pathogen‐specific humoral immune responses. Despite the advancements in mRNA vaccination, certain immunological elements require further enhancement to improve efficacy. Clinically approved mRNA‐LNPs vaccines are passively internalized by various somatic cells following injection, including B cells, muscle cells, CD4^+^ T cells, and tissue‐resident or recruited antigen‐presenting cells (APCs) [128]. Biodistribution studies indicate that the majority of intramuscularly injected mRNA vaccines are absorbed by local muscle cells, while the remainder enters the bloodstream, predominantly accumulating in the liver and spleen, which can lead to off‐target effects [129]. Therefore, specific delivery of mRNA‐LNPs to immune cells has the potential to strengthen and prolong immune protection, ultimately reducing the required dose of mRNA‐LNP and minimizing side effects. As previously mentioned, achieving this targeting involves employing ligands that specifically bind to cellular receptors located on the surfaces of the intended cells [130]. In recent years, researchers have been working to designing mRNA vaccines targeting APC, especially DCs, through in vivo delivery. Transporting mRNA to DCs boosts the presentation of antigens, consequently strengthening immune responses that fight against illness. Consequently, targeting DCs in situ represents a promising direction for the development of the next generation of mRNA vaccines. This approach could simplify the production process, reduce costs, and enhance vaccine safety. In a preclinical investigation, the overall charge of RNA‐liposomes was modified to improve the accurate targeting of DCs in the spleen, resulting in strong activation of memory and effector T cell responses that replicate the immunostimulatory pathway driven by Type I interferon (IFN). While specific antibodies or ligand‐conjugated LNPs have demonstrated DC specificity, their current application is limited to subunit vaccines [131, 132, 133], and evidence regarding their efficacy in mRNA vaccines remains scarce. The intercellular adhesion molecule 3‐grab nonintegrin specific to DCs (DC‐SIGN) functions as a pattern recognition receptor (PRR). When compared with nonspecific lentiviral vectors, the pseudotyped recombinant lentiviral vectors that use Sindbis viral glycoprotein as a ligand for DC‐SIGN demonstrate a potentially enhanced performance; however, the risk of insertional mutations poses a significant limitation to their clinical application. Researchers developed a virus‐like particle targeting DCs, which carries mRNA encoding SARS‐CoV‐2 spike protein or herpes simplex virus glycoproteins. Injection into mice enhanced immune responses and protection against both viruses, suggesting the potential for effective vaccines [134].

## 5 Key Considerations and Challenges

This section provides an in‐depth analysis of the persistent challenges faced by the mRNA‐LNP delivery system. It systematically examines three core bottlenecks: off‐target effects in nonliver tissues, vector immunogenicity, and insufficient endosomal escape efficiency. The existing solutions and corresponding research advancements are also summarized.

### 5.1 Delivery Precision

*Off‐target effects, hepatocyte dominance*: NPs exhibit a strong affinity for the liver after systemic administration due to the organ's unique physiological properties. These include abundant macrophages capable of uptaking NPs, a porous blood vessel structure that retains NPs, and a large blood volume with slow blood flow, which facilitates NPs interaction with liver cells [135]. This tropism poses significant challenges for delivering mRNA to nonliver organs after systemic administration. The preferential uptake of mRNA in the liver presents a significant challenge for the systemic delivery of mRNA to organs beyond the liver. Another critical issue in accurately targeting gene editing agents to specific cells is the presence of intrinsic biological barriers, such as the blood–brain barrier (BBB), which severely restricts vector entry into the body [136]. Currently, common methods employed to achieve targeted delivery include the use of specific recognition molecules (active targeting), LNP formulations that influence protein adsorption and alter in vivo biodistribution (endogenous targeting) [137, 138, 139], and modifications to the carrier's nature and the mode of administration (passive targeting) [138]. Based on these approaches, initial successes in the targeted delivery of cells have been achieved.

**FIGURE 3:**
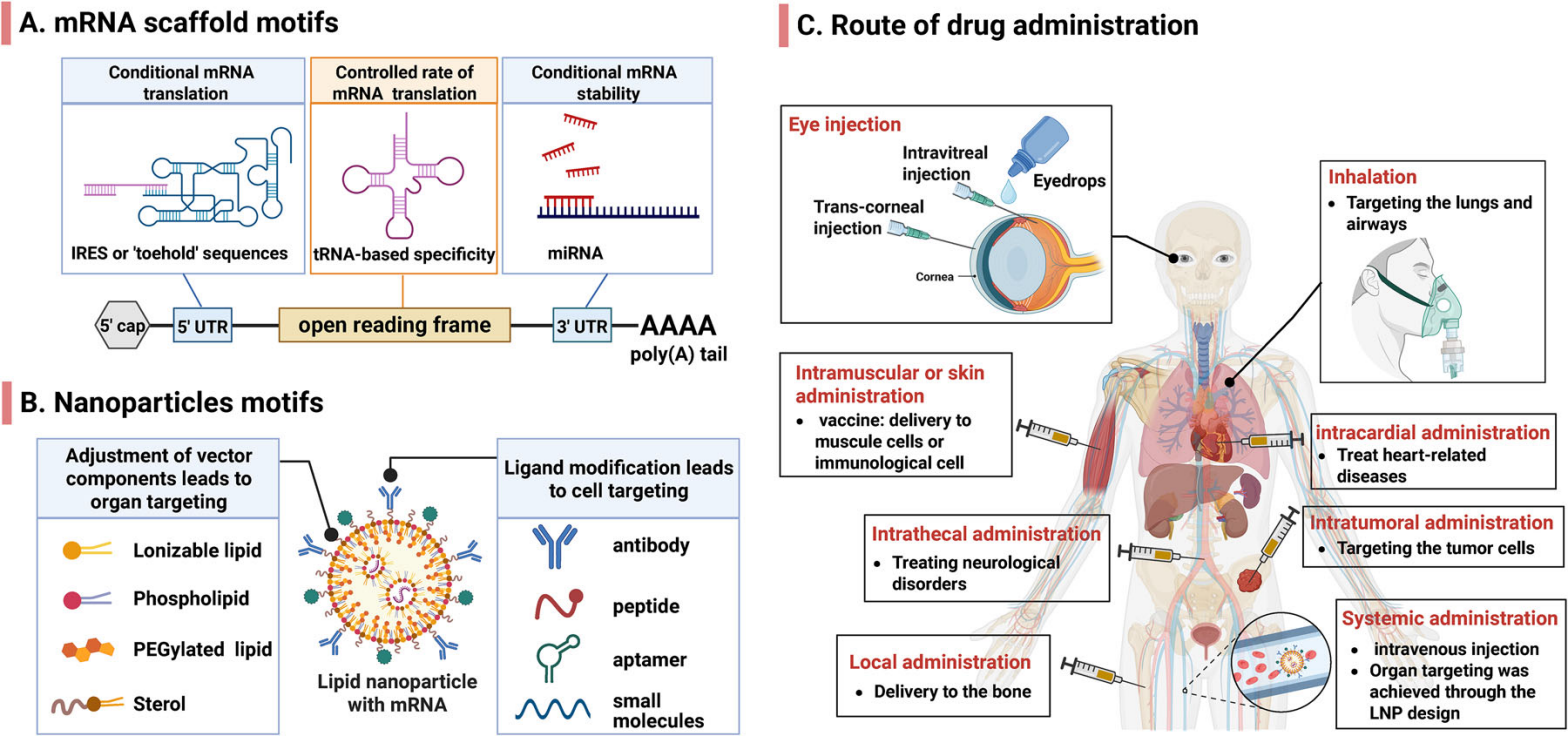
Cell‐targeting strategy for mRNA‐based delivery. (A) Conditional mRNA expression can be controlled by specific sequences within the mRNA. Strategies using reactive structures in the 5′ UTR and IRES regulate translation initiation, while unique tRNA patterns and cell‐specific miRNA binding sequences affect translation speed and degradation rates in different cell populations. (B) Active targeting methods employ targeting ligands to facilitate specific binding to cells that express target receptors, whereas passive targeting depends on the inherent properties of materials to direct them toward specific organs and cell types of interest. (C) Superficial surface targeting can be accomplished through local administration, whereas systemic administration necessitates the design of vectors to ensure specificity for extrahepatic organs and cells.

*Strategies for extrahepatic targeting*: Given these obstacles, it is both crucial and challenging to implement appropriate chemical modifications, optimize vector design, and select effective drug delivery strategies for the efficient and safe transfer of mRNA into target cells (Figure 3). Whether through the targeted design of the delivery vector or the enhancement of mRNA circulation stability, the primary goal is to improve the protein expression efficiency of mRNA, thereby ensuring the safety and effectiveness of mRNA drugs. Enhancing expression efficiency reduces the frequency of administration and mitigates toxic side effects and tolerance associated with repeated dosing. Several strategies have been developed to address these barriers, which will be discussed in the following sections.

#### 5.1.1 Route of Drug Administration

*Systemic administration* involves the administration of mRNA drugs through the bloodstream to various parts of the body. A significant challenge associated with this approach is ensuring that the mRNA can be specifically targeted to the desired tissue or cell. Currently, liver is the primary target organ for the systemic administration of mRNA, as it can internalize and eliminate circulating nanomaterials from circulation. In contrast, the efficiency of systemic delivery to other organs, such as the lung, spleen, muscle, skin, or tumors, varies significantly in efficiency and often necessitates the design and optimization of organ‐specific carrier materials. The current research on targeting strategies based on systemic delivery has always focused on the design and optimization of delivery materials, as will be detailed in the next chapter.

*Local delivery* entails direct administration of mRNA into the target tissue or organ, accomplished through methods such as lung inhalation (local inhalation) or intratumoral injection. This approach effectively circumvents several challenges like clearance and stability issues in the bloodstream, as well as nonspecific tissue uptake. Local delivery is applicable vaccination, tumor treatment, and bone regeneration. However, it is noteworthy that local administration is only indicated for known and accessible diseases at pathological sites.

*Intramuscular or skin administration*: The delivery of mRNA through intramuscular or skin injection is currently employed in specific vaccines and therapies requiring localized immune responses. Studies show that when mRNA is administered intramuscularly in “naked” or liposome‐encapsulated forms, protein expression predominantly occurs in muscle somatic cells [140]. A subset of DCs, known as Langerhans cells, located in the skin epidermis, suggests that intradermal administration might be be more effective than the traditional intramuscular or subcutaneous routs [141]. Enhancements in targeting APCs can be achieved through surface modifications of the carrier.

*Local inhalation administration*: Inhalation for delivering mRNA is primarily limited to lung tissue, making them an effective approach for treating lung cancer and certain respiratory diseases. Research indicates that both liposomes and polymer–matrix NPs can efficiently deliver mRNA to the lungs through inhalation [74, 142], with polymer–LNP formulations demonstrating further potential for development [143]. However, clinically used nebulizers often deform LNPs to deform due to the strong shear forces generated by compressed air or vibrating meshes, which can diminish the stability and transfection efficiency of mRNA‐LNPs [144]. Consequently, research in this area has concentrated on adjusting formulation and dosing parameters to enhance the delivery efficiency of mRNA during inhalation. Specifically, studies have shown that optimizing the molar concentration of polyethylene glycol (PEG) can protect LNPs from the aerosolization process, thus facilitating effective mRNA expression in the lungs [145, 146]. From a biological perspective, the inhalation delivery route encounters clearance mechanisms in the lungs, where particles are captured by mucus and expelled through ciliary motion from the deeper lung regions. To address this challenge, several parameters must be considered in particle design, including the use of smaller nanoscale particle sizes, maintaining an overall neutral charge to enhance particle design, including the use of smaller nanoscale particle size, maintaining an overall neutral charge to enhance particle diffusion in mucus, and incorporating excipient formulations that stabilize the particles. Additionally, designing moieties capable of binding to epithelial cell membranes is crucial [147]. Jiang et al. optimized the LNP formula by introducing the hydrophilic branched polymer excipient bPEG20K, which enhanced the charge stability of LNPs during the atomization process [148]. Concurrently, studies have indicated that to effectively penetrate deep lung tissue, larger particles (>5 µm) with lower densities (<0.4 g/cm^3^) are more effective than smaller, denser particles [149]. To predict the nucleic acid delivery efficiency of LNP based on their chemical composition, the research team developed and identified FO‐32 and FO‐35 lipids through the training of a neural network model known as LiON (lipid optimization using neural networks) [150]. The effective delivery of these lipids to ferret alveoli and conducting airways was demonstrated [150]. This finding indicates that deep learning can significantly aid in the optimization and design of LNP. Moving forward, there are opportunities to develop particulate systems that can penetrate deep into the lungs and subsequently release NPs [151], thereby enhancing the efficiency and therapeutic efficacy of inhaled mRNA delivery.

*Intratumoral administration*: Intratumoral administration is the directly way to deliver mRNA‐LNP to superficial tumors, and translation occurs even without a carrier. Tumors often have disorganized blood vessels, no lymphatic drainage, and dense extracellular matrix (ECM), helping retain these drugs [152]. Thus, research focuses on improving mRNA's therapeutic effect and carrier delivery efficiency. Numerous preclinical studies have focused on expressing specific cytokines and cytotoxins through direct intratumoral injection, as well as delivering CRISPR–Cas9 for oncogene targeting [153, 154, 155, 156, 157]. However, in practice, NPs are not confined solely to the tumor area; they are also distributed to the liver and lymphoid organs [152, 155], which may raise safety concerns regarding direct cytotoxic therapies, such as toxins and cell cycle regulators. Furthermore, mRNA is primarily expressed by immune cells, particularly macrophages, rather than being restricted to cancer cells [154, 155]. Therefore, intratumoral injection therapy is generally appropriate when the target protein is tolerated by surrounding nonmalignant tissues and is often employed in conjunction with immune checkpoint inhibitors [158]. Studies have also found that the local expression of certain cytokines can mitigate the dose‐limiting adverse events associated with systemic administration [153]. Consequently, the current clinical stage of mRNA‐LNP intratumoral injection candidate drugs primarily focus on strategies for encoding IL‐12. This approach offers a means to enhance antitumor immune responses while facilitating the local expression of highly toxic cytokines by manipulating the TME. However, its application is restricted to palpable solid tumors and necessitates tolerance in nonmalignant tissues in addition to cancer cells.

*Targeted local administration in specific tissues*: Taking advantage of the unique physiological barriers and retention properties of specific tissues, precise targeted delivery can be achieved through local injection. This strategy minimizes systemic exposure and is categorized below by tissue type:

1. *Bone Regeneration*: In the realm of orthopedics, local delivery circumvents the poor perfusion of bone tissue. For instance, BMP‐2 mRNA delivered directly into bone defects using a C12‐ionizable lipid formulation induced robust protein expression. Quantitative analysis revealed a significantly enhanced bone regeneration rate, with treated groups showing complete bridging of critical‐sized defects by 4 weeks compared with controls [105]. This demonstrates that local retention of mRNA‐LNPs can drive structural tissue repair.
2. *Ocular Delivery*: The eye is an enclosed compartment ideal for local therapy. Subretinal injection of mRNA to retinal pigment epithelial cells has been used to address genetic disorders [159]. Overcoming the corneal barrier remains a challenge; however, Zhou et al. developed exosome‐encapsulated mRNA (MSC‐exo) eye drops. This noninvasive approach successfully penetrated the corneal barrier, significantly ameliorating dry eye disease symptoms by restoring miR‐204 expression levels [160].
3. *Cardiac Repair*: Intracardiac injection allows for high local concentrations in the myocardium. Naked VEGF‐A mRNA injection has been shown to improve cardiac function following myocardial infarction, significantly increasing left ventricular ejection fraction by approximately 6.6% and reducing infarct size [102]. Regarding expression kinetics, Turnbull et al. used lipid nanocomplexes to deliver mRNA, detecting translated protein expression persisting for 2–4 days postadministration [161]. Similarly, Singh et al. utilized alginate hydrogels to encapsulate mRNA, observing specific protein expression restricted to myofibers for 3–4 days, with minimal leakage to off‐target organs [162]. These data suggest that local matrices can effectively retain mRNA at the injection site.
4. *Central Nervous System*: The BBB severely limits systemic delivery. Intrathecal and intracerebral routes allow mRNA to bypass this barrier. For example, intrathecal delivery of mRNA‐LNPs has demonstrated the ability to induce protein expression in dorsal root ganglia, offering a potential treatment for Friedreich's ataxia [163]. While this route achieves high local expression with reduced systemic toxicity, its invasive nature limits the feasibility of repeated dosing. Future research focuses on extending the half‐life of mRNA to reduce administration frequency.

#### 5.1.2 Targeted Delivery of mRNA Based on NP Motifs

Due to the chemical and physical properties of biological macromolecules and their negative charges, naked mRNA cannot effectively enter cells. Recently, multiple NPs have been created to deliver mRNA, categorized as viral and nonviral vectors. Due to immunogenicity and toxicity, most mRNA delivery uses nonviral vectors like LNPs, peptide‐based nanovesicles, lipid‐polymer hybrids, exosomes, polymers and so on. Currently, LNPs represent the mainstream clinically proven platform for RNA therapy delivery [164]. Encapsulation by LNPs stabilizes RNA against nuclease degradation and prevents its rapid clearance from the bloodstream. Furthermore, the targeting capability to specific cell types can be enhanced by altering the surface properties, size, and charge of the LNPs or by increasing the modification of the carrier surface (Figure 3B). For instance, immune cell receptor‐modified LNPs can enhance the uptake of mRNA therapeutics by specific types of immune cells. Subsequent sections discuss advancements in targeting specific cells through the modification of LNP properties and surfaces in detail.

#### 5.1.3 Optimizing Components of LNP

Upon entering systemic circulation, NPs rapidly adsorb serum proteins, which can alter their transport and internalization pathways of LNPs [165, 166]. According to the Vroman effect, NPs initially adsorb high‐abundance proteins, such as albumin and fibrinogen, forming a soft layer. Subsequently, low‐abundance, high‐affinity proteins dynamically adsorb and exchange on the NP surface, leading to the formation of a hard layer [167, 168]. This process ultimately results in the establishment of a biomolecular corona characterized by a hard layer on the inside and a soft layer on the outside [169, 170]. Recent studies have demonstrated that modifying the surface properties of LNPs—such as their size, shape, surface charge, degree of polarization, and chemical characteristics—can alter the component of the biomolecular corona, thereby influencing the targeted distribution of LNPs. Furthermore, the formation of a biomolecular corona may impede the interaction between active targeting ligands on NPs and their target cells [171]. In contrast, for “passive” NPs lacking surface ligands, the biomolecular corona can bestow new functionalities and facilitate specific targeting capabilities.

Surface charge significantly influences the component of the biomolecular corona. Cheng et al. developed an organ‐selective targeting NP platform (SORT). This SORT delivery system incorporates cationic lipids, such as DOTAP, and anionic lipids, such as 18PA, into the widely utilized four‐component LNP system [138]. Additionally, the inclusion of an ionizable lipid as a fifth component alters the overall apparent p*K*_a_ value, leading to the distinct types formation of of protein corona on the surface of SORT LNPs. This modification facilitates the delivery of mRNA to the spleen and lungs of mice selectively, and potentially to the liver as well. Specifically, when the apparent p*K*_a_ of SORT‐LNPs is less than 6, the protein corona is enriched with β2‐glycoprotein I, which correlates with spleen targeting; when the p*K*_a_ is between 6 and 7, ApoE adsorption is associated with liver targeting; and when the pKa exceeds 9.25, vitreous mucin is enriched, facilitating lung targeting [5, 137]. However, several critical challenges remain for this technology. First, there is an immunogenicity risk associated with the modified lipids; cationic lipids commonly used in SORT are positively charged at physiological pH and can be recognized by in vivo PRRs (such as TLR4), activating innate immune responses and stimulating macrophages to secrete inflammatory cytokines like TNF‐α and IFN [44];Similar to the accelerated blood clearance (ABC) effect triggered by anti‐PEG antibodies in PEGylated LNPs [125], long‐term or repeated use of SORT‐modified lipids may induce lipid‐specific antibodies, leading to rapid clearance and reduced therapeutic efficacy. Furthermore, endogenous targeting relies on the formation of a protein corona, the composition of which is dynamic and undergoes remodeling with changes in the biological environment (e.g., protein concentration, pH, ionic strength) [172]. Finally, clinical translation faces hurdles regarding the metabolic pathways of novel degradable lipids, specifically whether degradation products accumulate in the liver or kidneys and the lack of long‐term safety data. Industrial purity control (e.g., removal of unreacted lipid monomers) also remains a technical challenge to meet strict regulatory standards for nanomedicines.

The chemical nature of lipid excipients can influence the composition of the biomolecular corona. While this alteration has minimal impact on the size and surface charge of NPs, it may significantly affect their interactions with proteins [173]. A recent study demonstrated that substituting amide bond‐modified ionizable lipid tails (N‐LNPs) with ester‐bond‐modified ionizable lipid tails (O‐LNPs) modifies the protein corona adsorbed on the surface of LNP components. Although albumin remains the most abundant protein in the biomolecular corona, the next most prevalent protein shifts from ApoE and complement C1 to fibrinogen beta and gamma chains, leading to a change in LNP targeting from the liver to the lung [173].

Inspired by the ability of neurotransmitters, such as tryptamine derivatives, to traverse the BBB, Ma et al. developed LNPs derived from neurotransmitter‐based liposomes (NT‐lipidoids). This innovation achieved effective brain delivery [174]. Their work expands the potential applications of LNPs to various organs and offers insights for targeted delivery to additional tissues. In a related study, Cai et al. designed ROS‐degradable ionizable lipids by incorporating thioketal moieties, thereby enabling selective mRNA delivery to tumor cells [175]. Xue et al. developed ionizable lipids functionalized with bisphosphonate (BP), producing BP‐LNPs that enhanced mRNA delivery within the bone microenvironment [176]. Additionally, Miao et al. synthesized liposomes featuring heterocyclic amine head groups, which activated immune cells and significantly enhanced the antitumor efficacy of the mRNA‐LNPs cancer vaccine [177]. Collectively, these examples underscore the importance of conducting in‐depth studies on structure–activity relationships to expedite the development of functional ionizable lipids for targeted applications.

Numerous researched have shown that particle size influences the composition of protein corona. For instance, 70 nm NPs with a small radius of curvature tend to adsorb small molecular proteins, whereas 200 nm NPs are more prone to adsorb larger molecular proteins [178, 179]. However, there is a lack of research examining the impact of NP size on organ targeting through its effect on the biomolecular corona. Nonetheless, some studies indicate that particle size does affect organ targeting. For example, mesoscale NPs with a particle size of approximately 400 nanometers can selectively aggregate in the kidney, particularly in the proximal renal tubules [180, 181]. In contrast, micron‐sized particles (microparticles) have been shown to accumulate more readily in the lungs [182, 183]. The specific mechanisms underlying the selective influence of particle size on target organs warrant further investigation.

#### 5.1.4 Surface Modification of LNP

**FIGURE 4:**
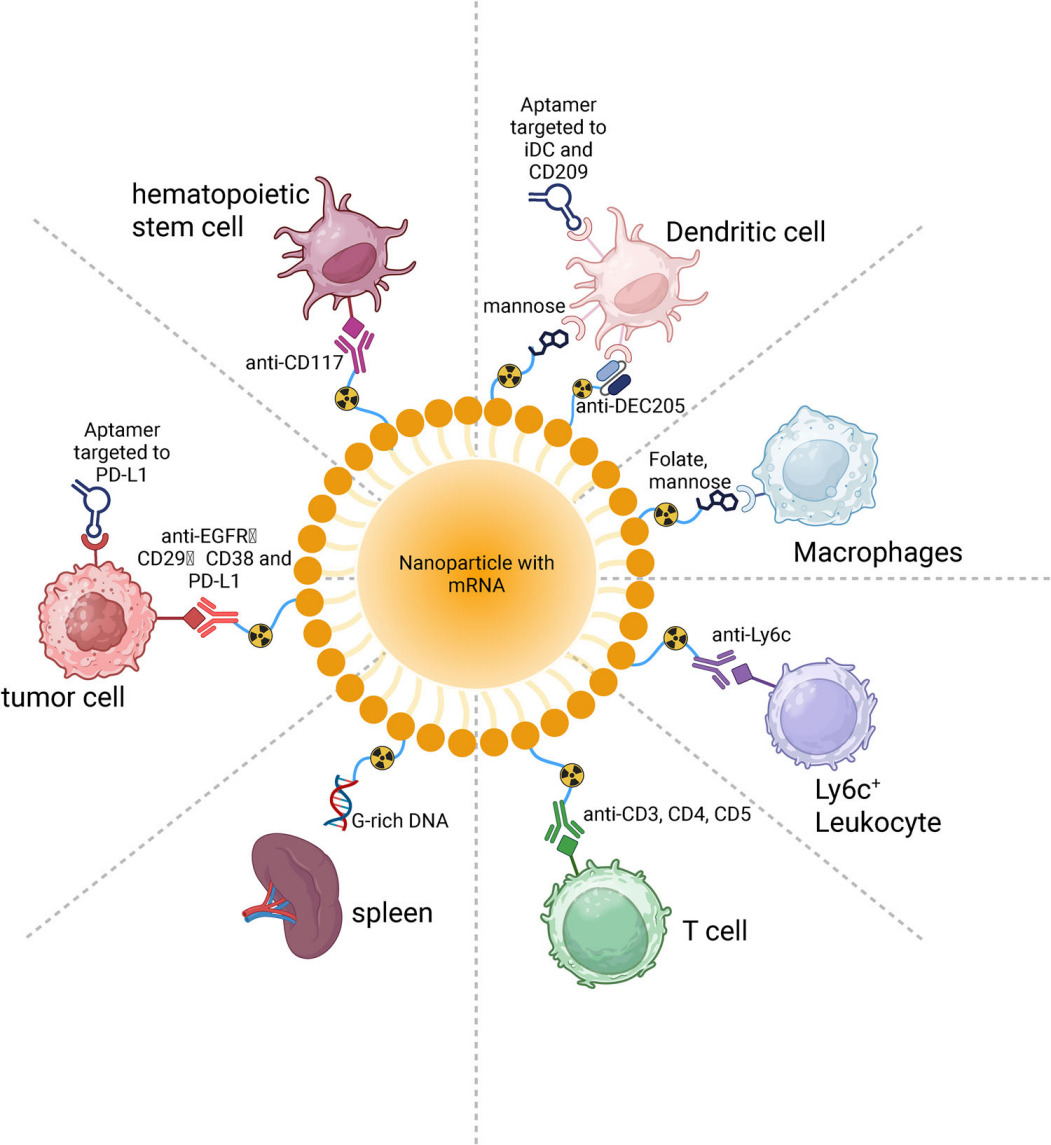
Vector modifications for mRNA delivery to specific cells. The primary modifications include peptides, small molecules (folate and mannose for macrophages, mannose for dendritic cells), antibodies (anti‐CD117 for hematopoietic stem cells, anti‐EGFR, CD29, CD38, and PD‐L1 for tumor cells, anti‐CD3/CD4/CD5 for T cells, anti‐Ly6c for Ly6c+ leukocytes, anti‐DEC205 for dendritic cells), DNA (G‐rich DNA for spleen cells), aptamers (PD‐L1 for tumor cells, iDC and CD209 for dendritic cells), and other relevant entities to achieve precise targeting in mRNA delivery to specific cells.

Modifying targeting ligands, such as small molecule inhibitors, peptides, antibodies, and nucleic acid aptamers, on the surface of LNPs can enhance the affinity of the carrier for target cells or tissues and improve targeting efficacy (Figure 4). This strategy, known as active targeting, is mainly achieved through three surface modification methods: first, the presynthetic ligand incorporation method, which integrates functionalized lipids containing targeting materials into the LNP assembly process via hydrophobic interactions, or inserts them into preprepared LNPs [184]; second, the in situ covalent modification method, which uses bioconjugation chemistry to covalently attach targeting molecules to the surface of preprepared LNPs [184]; and third, the electrostatic coating method, which achieves noncovalent modification via electrostatic interactions between charged targeting materials and LNPs [185, 186]. These methods can promote the cellular uptake of mRNA‐LNPs, which is crucial for the function of nucleic acid payloads.

First, it is necessary to identify the target, which involves considering the specific receptor present on the target cell. Designing a targeting ligand on the carrier's surface can enhance the loading efficiency of mRNA and minimize off‐target effects. Second, when evaluating potential targets, the action of the target ligand and the possible biological consequences of their interaction must be addressed [114]. For instance, mRNA‐LNPs targeting CD3 and are intended for delivering mRNA payloads to T cells have demonstrated adverse effects, including activation and depletion of circulating and splenic T cell subsets, irrespective of the payload's activity [114]. Additionally, the impact of ligand binding on internalization should be considered. For instance, small molecules such as folic acid (FA) trigger receptor‐mediated endocytosis (RME) upon binding to folate receptors, thereby altering the internalization pathway [187]. Similarly, peptides like MH42, upon binding to neuronal receptors, may modulate the fluidity of the endosomal membrane, facilitating endosomal escape [188]. mRNA therapeutics need to reach the cytoplasm and be translated into proteins to fulfill their intended functions. Thus, internalization and endosomal escape are critical for the effectiveness of mRNA‐LNPs. Furthermore, while ensuring successful payload delivery, antibodies that fail to internalize may activate effector functions, such as antibody‐dependent cellular cytotoxicity and other Fc region‐mediated activities in macrophages [189, 190]. Most information about the connection between targeting and receptor internalization comes from studies on antibody–drug conjugates, which serve as a valuable references. Consequently, a thorough biological comprehension of the targeting interaction, its disease context, and empirical evidence is essential for designing effective targeting strategies, beyond mere screening.

*Antibody motifs*: Targeting immune cells represents one of the most promising avenues for advancing original cell therapy. Evidence shows that LNPs modified with specific antibodies can achieve DC specificity [191]. To date, antibodies targeting CD3 [114], CD4 [113], and CD5 [112] have been utilized to modify LNPs for the in vivo delivery of mRNA to T lymphocytes, demonstrating marked improvements over nontargeting mRNA‐LNPs. Notably, antigen expression in CD4^+^ T cells, targeted by modified mRNA‐LNPs, increased by a factor of 33 [113]. Veiga et al. developed anti‐Ly6c modified LNPs for targeted delivery to Ly6c^+^ leukocytes in vivo [192]. In comparison with unmodified LNPs, they observed a significant increase in antigen expression in target organs and a significant decrease in the liver [192]. This evidence demonstrates that antibody‐based targeting can achieve a high degree of cell specificity. In the realm of stem cells, Breda et al. decorated mRNA‐LNP with an antibody against the HSC membrane receptor c‐KIT (also known as CD117), successfully facilitating effective delivery to mouse HSCs in vivo, which lays the groundwork for the realization of gene editing in stem cells [193]. Specific antibodies for tumor cells include EGFR [156] and CD44 [194, 195]. Overall, antibody‐based modifications hold significant potential for enhancing cell specificity in targeting.

*Other motifs*: Compared with antibodies, small molecules and peptides are often easier to manufacture and translate from animal models to human applications. Instances of peptide targeting elements consist of iNGR and iRGD peptides, which facilitated efficient delivery into bone metastases and solid tumors via lipid conjugation [196, 197, 198, 199]. Herrera‐Barrera et al. produced LNPs modified with the photoreceptor targeting peptide MH42, enabling mRNA delivery to the retinal pigment epithelium for the treatment of hereditary retinal degeneration [188]. Given that the folate receptors are highly expressed on various tumor cells, researchers designed FA modified LNP to explore FA functionalization for tumor targeting [187]. Additionally, Kang et al. designed a polyethylenimine modified with mannose receptors to specifically target macrophages [200]. Nucleic acid aptamers have emerged as optimal drug‐targeting ligands due to their ability to specifically bind to receptors on the surface of cell membranes, thereby facilitating the endocytosis of NPs into cells. The authors coupled iDC and CD209 nucleic acid aptamers to NPs, resulting in the construction of an aptamer‐functionalized nanovaccine capable of specifically targeting circulating classic DCs in vivo [201]. Sam Lee et al. designed a LNP that binds to an anti‐PD‐L1 DNA aptamer, enabling the delivery of PTEN‐encoding mRNA to CRPC expressing PD‐L1 [202]. Furthermore, DNA‐modified LNPs (LNP‐SNAs) decorated with G‐rich motifs allowed for targeted delivery to the spleen via G‐quadruplex‐mediated endocytosis through serotonin receptors [203]. These findings suggest the potential for various modifications, including antibodies, peptides, and aptamers, in enhancing the targeting capabilities of mRNA‐LNPs.

#### 5.1.5 mRNA Scaffold Motifs

In addition to the delivery vehicle, it is crucial to investigate the mRNA cargo itself, as the mRNA molecule serves as the effector in mRNA‐LNPs drug. Regulating the selective expression of mRNA in specific tissues and cells could offer a promising approach to enhance tissue or cell specificity(Figure 3A). Recent studies have demonstrated that the introduction of siRNA target sites into the Cas9 mRNA backbone can reduce off‐target editing in liver cells, thereby enhancing the specificity of LNP‐mediated gene editing in the spleen and lung [204]. Given the cellular heterogeneity of endogenous microRNAs (miRNAs), such as miR‐122 in the liver and miR‐142 in the spleen, the introduction of specific miRNA target sites (binding sequences) into mRNA sequences can effectively inhibit the expression of target mRNA in unintended cells [205, 206, 207]. For instance, the insertion of miR‐142 target sites into mRNA resulted in reduced expression levels in spleen cells, while insertion of miR‐122 target sites led to diminished expression in the liver [206]. This approach enhances the control of protein translation in specific cell types within target tissues. Importantly, endogenous miRNAs do not require additional delivery vehicles, making them easier to manipulate [208]. Recently, Qiang Cheng et al. have developed a SELECT approach (Simplified LNP with Engineered mRNA for Cell‐type Targeting), which effectively differentiates target tissues or cells based on the abundance of intracellular miRNA [208]. This strategy improves the precision treatment of tumor cells.

Additionally, sequences that can be programmed have been utilized for translation specific to certain cells. The activation of translation can be facilitated by altering the structural dynamics of the mRNA molecule. For instance, “toehold” sequences located in the 5′ UTR of the mRNA inhibit translation unless they are bound by target cell‐specific transcripts [190]. Moreover, inducible internal ribosome entry site (IRES) sequences can be used to control the initiation of translation, allowing for more precise regulation through structural alterations in the mRNA [209, 210]. Furthermore, differential expression can also be influenced by codon choice within the open reading frame. This strategy leverages the nonrandom distribution of tRNA expression across different cell types and tissues, enabling mRNA payloads to achieve cell‐specific expression levels [211, 212, 213]. Consequently, the expression level of the mRNA payload can also achieve cell specificity.

### 5.2 Safety and Immunogenicity

#### 5.2.1 Innate Immune Sensing, Cytokine Response

The application of mRNA‐LNPs may activate the innate immune system, leading to unintended immune responses. This occurs primarily through PRRs, such as TLRs [214] and retinoic acid‐inducible gene I‐like receptors [215]. Single‐stranded RNA activates TLR7 and TLR8, while double‐stranded RNA (dsRNA) activates TLR3. Triggering these receptors initiates immune signaling cascades. Although this effect can be beneficial in vaccine applications by enhancing immunogenicity, it often causes inflammatory responses in gene editing or protein replacement therapies. Such activation promotes the production of proinflammatory cytokines via NF‐κB or IRF3 pathways [216], which can suppress mRNA translation in vivo [217] and significantly compromise therapeutic efficacy. Furthermore, chronic immune activation in long‐term treatments may lead to immune‐mediated damage to target cells, reducing their survival and function. One study also indicated that the release of inflammatory cytokines and chemokines during mRNA‐LNP treatment may originate from the LNP components themselves [218]. The inflammatory properties of CILs can further initiate immune cascades [219], highlighting that immunogenicity related to the LNP carrier cannot be overlooked. Additionally, dsRNA by‐products generated during in vitro transcription can activate TLR3, leading to TIR‐domain‐containing adapter molecule 1‐mediated production of Type I IFNs [216]. This triggers systemic immune responses and cytokine release. In protein replacement therapies, dsRNA‐dependent enzyme activation can inhibit protein synthesis and impair treatment outcomes [220].

#### 5.2.2 Mitigation via Nucleoside Modification, Purification

To reduce TLR recognition during in vivo delivery, nucleoside modifications of mRNA sequences can be employed [221, 222]. Several chemical modifications—including 5‐methylcytidine (m5C), N6‐methyladenosine (m6A), 5‐methyluridine (m5U), 2‐thiouridine (s2U), and pseudouridine (Ψ)—have been applied to in vitro transcribed mRNA, as summarized in Table 5. Seminal work by Karikó et al. demonstrated that Ψ‐modified mRNA reduces IFN responses and inflammatory cytokine production [223]. Nucleoside‐modified mRNA also exhibits extended translation duration and half‐life [224], indicating that such modifications can enhance the therapeutic efficacy of mRNA applications. Similarly, Mokuda et al. confirmed that m1Ψ‐modified mRNA enhances protein expression while reducing cytokine expression [225]. Notably, the COVID‐19 mRNA vaccines mRNA‐1273 (Moderna) and BNT162b2 (BioNTech/Pfizer) utilized m1Ψ to fully replace uridine [226]. In practice, these modifications are often used in combination to optimize performance. For example, one study showed that comodification with m1Ψ and m5C synergistically reduced cytokine levels and minimized immune‐related interference [227]. Removing dsRNA contaminants through further purification of in vitro transcribed mRNA can also improve translation efficiency and limit cytokine induction. Simple and cost‐effective methods such as cellulose‐based chromatography are suitable for small‐scale production, though they require high‐salt conditions and have limited throughput [220]. For industrial‐scale vaccine production, BioNTech/Pfizer employs a patented IP‐RP‐HPLC technique that offers high resolution. Alternative methods include tangential flow filtration for size‐based separation and enzymatic degradation of dsRNA into small fragments for removal. Purification of the final mRNA‐LNP complex is equally critical. The choice of purification method should be aligned with the LNP preparation process—such as surface modification and the presence of composite carriers—and the intended application. Dialysis is suitable for initial purification of conventional LNPs, ultrafiltration is effective for surface‐modified LNPs, and size exclusion chromatography or gel filtration is ideal for high‐precision requirements. Proper implementation of these purification steps significantly reduces unintended side effects after in vivo administration, such as hemolysis caused by free lipids or immune activation by unencapsulated mRNA, while maintaining the structural integrity and targeting function of mRNA‐LNPs [228, 229].

**TABLE 5:** Lipid components and the function in LNPs.

Modifications of mRNA sequences	Function	Application
Pseudouridine (Ψ)	Reduce immunogenicity and enhance expression	The first modified nucleoside to reduce immunogenicity effectively [223]
N1‐methylpseudouridine (m1Ψ)		COVID‐19 vaccines from BioNTech (COMIRNATY) and Moderna (Spikevax)
5‐Methylcytidine (m5C)		It is often combined with other modifiers such as Ψ [227]
5‐methyluridine (m5U)		One of the modified protocols explored in the study was better than unmodified uridine, but not as good as pseudouridine
N6‐methyladenosine (m6A)	Endogenous regulation	More commonly seen in basic research
2‐Thiouridine (s2U)	Improve translation accuracy	As an adjuvant therapy it does not cause alone

### 5.3 Expression Kinetics

The duration of protein expression mediated by mRNA‐LNPs can be tuned from hours to days by adjusting mRNA structure and LNP composition, allowing alignment with specific therapeutic windows. However, precise control over expression kinetics remains challenging and requires optimization of mRNA design, LNP formulation, and administration routes. Unmodified mRNA is susceptible to recognition by the innate immune system and rapid degradation, while excessively stabilized structures may lead to unduly prolonged expression. Therefore, a balance must be achieved between translation initiation rate and duration. Current strategies include using CAP1 structures to maintain translational efficiency while avoiding rapid degradation induced by immune recognition [230]. Controlling mRNA half‐life is particularly important for timed degradation. This can be accomplished by incorporating specific binding sites or regulatory elements within the UTRs to enable active regulation of mRNA degradation within cells.

The composition and physicochemical properties of LNPs significantly influence the rate of mRNA release in vivo. Rapid release enables quick but transient expression, while slower release supports sustained protein production. Adjusting the ratios of LNP components or incorporating auxiliary lipids can enhance NP stability and modulate endosomal escape kinetics. For example, Sabnis et al. developed novel amino acid‐based lipids that improve endosomal escape and enable efficient mRNA delivery [231], providing valuable insights for future LNP optimization. Furthermore, expression kinetics are influenced by the metabolic and endocytic rates of different organs and cell types. Finally, the route of administration fundamentally determines expression profiles by defining the physiological environment encountered by LNPs, the target cell types, and the rate of lymphatic drainage. Intramuscular or subcutaneous injection, commonly used for vaccines, promotes rapid local transfection and high‐level expression. Intravenous administration is better suited for secretory protein therapies requiring sustained systemic expression. Studies have demonstrated that different administration routes lead to distinct pharmacokinetic profiles, underscoring their decisive role in shaping expression kinetics [232].

## 6 Future Directions

As the core delivery system for mRNA therapeutics, LNPs have shown significant potential for clinical translation. However, to meet the diverse demands of complex disease treatment and large‐scale application, next‐generation LNPs are expected to achieve breakthroughs in functional design, development paradigms, and translational capabilities.

### 6.1 Innovations in Functional Design: Toward Precision and Intelligence

The core pursuit of next‐generation LNPs lies in enhancing delivery precision, functional adaptability, and clinical accessibility to overcome the inherent limitations of traditional systems.

#### 6.1.1 Precision Targeting: From Organ‐Level to Cell Subtype Specificity

Targeted delivery is crucial for improving therapeutic efficacy and reducing off‐target toxicity. While established strategies have enabled preliminary organ‐level targeting for LNPs, achieving precise delivery to disease‐specific cell subtypes remains a key bottleneck. For instance, cystic fibrosis is caused by mutations in the cystic fibrosis transmembrane conductance regulator (CFTR) located on the apical membrane of respiratory epithelial cells, making these cells ideal therapeutic targets. However, the specific molecular mechanisms regulating LNP recognition and binding to these target cells are not yet fully elucidated, highlighting the need for in‐depth exploration of cell subtype‐specific targeting motifs and interaction pathways.

#### 6.1.2 Bio‐Inspired Intelligence: Stimuli‐Responsive Logic Control

Traditional LNP research has often focused on single components or isolated functions, overlooking the dynamic, integrated behavior of LNPs as a delivery system. Next‐generation LNPs will adopt bio‐inspired design principles, integrating various stimuli‐responsive elements (e.g., pH‐sensitive, enzyme‐sensitive, photo‐sensitive) to perform AND/OR/NOT logic operations, enabling activation specific to the pathological site. For example, researchers are developing LNP systems capable of synergistic responses to multiple TME signals, releasing anticancer drugs only upon simultaneous detection of tumor‐specific signals [229]. This design prevents premature drug release in healthy tissues, significantly enhancing treatment specificity and, consequently, clinical safety.

#### 6.1.3 AI‐Driven Rational Design: Accelerating LNP Optimization

Ionizable lipids are the core of LNP formulations, determining NP potency and organ tropism, and have been extensively studied for mRNA delivery. AI has emerged as an effective tool for predicting rational LNP compositions. A research team at MIT developed a deep learning‐based LNP design system (the AGILE platform). By analyzing structure–property data from thousands of LNP formulations, the system learned to predict transfection efficiency, leading to the discovery of H9 (an LNP with several‐fold higher transfection efficiency than conventional formulations) and R9 (a macrophage‐specific targeting formulation)—breakthroughs that could have taken years using traditional methods [233]. Subsequently, the TransLNP system, built based on the Transformer architecture, further improved prediction accuracy [234]. Beyond predicting transfection efficiency, TransLNP can also decipher interaction mechanisms between lipid molecules, providing actionable guidance for designing novel lipid components. However, limitations remain: the model heavily relies on in vitro data, lacks sufficient predictive precision for the complex in vivo environment, has weak interpretability, and does not adequately incorporate synthesis feasibility and toxicity assessment. In the future, such platforms need to evolve toward multidimensional data integration, incorporating data on in vivo fate and specific cell types, while also leveraging molecular dynamics simulations to enhance interpretability.

### 6.2 Advances in Translational Application: Scalability, Accessibility, and Stability

For mRNA‐LNP therapies to fully realize their clinical potential, three core issues must be addressed: scalable manufacturing, process simplification, and enhanced stability.

#### 6.2.1 Scalable Production: Overcoming Industrialization Bottlenecks

Producing high‐quality LNPs with batch‐to‐batch consistency at scale remains a major challenge. While microfluidic technology excels at laboratory‐scale synthesis, its industrial scale‐up faces numerous technical bottlenecks. To meet commercial demands, several companies are developing specialized manufacturing equipment and processes tailored for LNP production, aiming to bridge the gap between lab‐scale innovation and scaled clinical supply. Furthermore, increasing mRNA transfection efficiency to reduce the required dose has become a complementary strategy for improving scalability.

#### 6.2.2 Process Simplification

Current industrial mRNA‐LNP production processes remain relatively complex and costly. Recently, to improve the accessibility of mRNA‐LNPs, researchers have developed a novel “postencapsulation” preparation method characterized by extreme simplicity and “mix‐and‐use” convenience. This method involves preforming “empty” LNPs (without mRNA) and formulating them into a stable, ready‐to‐use liquid suspension (RtoU/Liq) [235]. When needed, functional mRNA‐LNPs are rapidly formed by simply mixing the premade empty LNP suspension with the target mRNA solution. Simplifying the mRNA‐LNP preparation process significantly lowers the barrier to mRNA drug development, accelerating global mRNA research and translation.

#### 6.2.3 Stability Optimization: Achieving Room‐Temperature Storage

Current mRNA‐LNP formulations have poor liquid stability, requiring storage at ultra‐low (−90 to −60°C) or low (−50 to −15°C) temperatures, which imposes significant logistical burdens and cost pressures on the global distribution, storage, and administration of vaccines. Recently developed novel continuous lyophilization technology addresses this limitation: this technology enables long‐term storage of mRNA‐LNPs at room temperature or even 37°C without loss of biological activity. This breakthrough is transformative—it allows mRNA vaccines to be transported and stored under standard conditions like traditional vaccines, significantly reducing costs and waste while improving accessibility [236]. Therefore, enhancing the exploration of room‐temperature stable mRNA‐LNP formulations strengthens global preparedness for future pandemics and expands the clinical potential of the entire mRNA therapeutics field.

## 7 Conclusion

In recent years, the rapid advancement of mRNA technology has demonstrated significant potential. Cell engineering utilizing mRNA technology represents a significant advance in the field of cell therapy. Moreover, the greater promise lies in the ability to engineer in vivo cells through mRNA technology. This approach to editing in situ cells could reduce costs and facilitate numerous applications that are not feasible with in vitro cell engineering. Nevertheless, the broad implementation of mRNA technology in vivo continues to encounter several challenges.

The first is to overcome the physiological environment in vivo to achieve targeted delivery. Some promising progress has been made in targeted delivery of mRNA‐LNPs via passive or active strategies. However, the delivery of LNPs still faces challenges, including toxicity from accumulation in the liver and spleen, difficulties in organ‐selective delivery, pharmacokinetics, and immune responses, necessitating ongoing research and optimization. The selection of a targeting strategy must take into account factors such as the establishment of a targeting profile, the feasibility of local injection, and the safety of the vector for in vivo delivery. For instance, the incorporation of cationic or anionic lipids can facilitate the targeting of LNPs to extrahepatic organs; however, these lipids may exhibit toxicity, which restricts their clinical applicability [237]. Further attention should be paid to the variety of payloads available, the significance of their off‐target toxicity, their specific expression for therapeutic efficacy, and their manufacturability. The selection of any particular method often entails a compromise between production ease and specificity. Additionally, it may be promising to explore methods for the selective expression of mRNA in target cells by modifying and optimizing the sequence or structure of the mRNA. Furthermore, it is essential to assess the quantity of protein that must be translated by mRNA to attain therapeutic levels across various applications within the body. For instance, protein replacement therapies and vaccines necessitate prolonged expression of cellular mRNA drugs, which in turn demands enhancements in the stability and duration of mRNA expression. Additionally, individual variability may result in different protein requirements, making it prudent to screen candidate mRNAs that encode effective proteins with a broad therapeutic window at low doses.

Looking ahead, in vivo delivery based on mRNA should be safe, effective, manufacturable, and affordable. We anticipate the development of diverse methods for achieving specific cell engineering in the future, which could lead to new therapeutic applications for a wider range of diseases.

## Author Contributions

Tian Xie provided the framework of the review. Lina Li played a pivotal role in the drafting and development of the manuscript. Menglan Wang and Xiuhan Ye was responsible for compiling the tables and creating the figures that were included in the manuscript. Zhiyan Liu and Yijing Duan worked together to gather and organize the relevant literature and data, ensuring that the manuscript was well supported by existing research. Xiaoming Chen and Yue Ouyang assisted in revising the manuscript. Qibiao Wu and Mengjuan Sun then reviewed and edited the manuscript, making necessary corrections and improvements to enhance its clarity and accuracy. All authors have read and approved the final manuscript.

## Funding

This study was funded by the Key Project of Social Development of Jinhua Science and Technology Bureau of Zhejiang Province, China (No. 2023‐3‐165; No.2022‐3‐137; No.2024‐3‐042), Basic scientific research plan of Hangzhou Medical College (KYQN2024008), Natural Science Foundation of Zhejiang Province (No. LTGY23H070001), and Science and Technology Development Fund, Macau SAR (0048/2023/AFJ and 0164/2023/RIA3).

## Ethics Statement

The authors have nothing to report. No datasets were generated or analyzed during the current study. This review article does not involve any studies with human participants or animals performed by any of the authors.

## Conflicts of Interest

The authors declare no conflicts of interest.

## Data Availability

The authors used ChatGPT 4.0 to assist with literature sorting and language polishing during manuscript preparation. All core academic content is original and has been verified by the authors for scientific accuracy.
